# The Molecular Mechanisms of Zinc Neurotoxicity and the Pathogenesis of Vascular Type Senile Dementia

**DOI:** 10.3390/ijms141122067

**Published:** 2013-11-07

**Authors:** Dai Mizuno, Masahiro Kawahara

**Affiliations:** Department of Bio Analytical Chemistry, Research Institute of Pharmaceutical Sciences, Musashino University, Nishitokyo-shi, Tokyo 202-8585, Japan; E-Mail: makawa@musashino-u.ac.jp

**Keywords:** Zinc, carnosine, histidine, vascular-type dementia

## Abstract

Zinc (Zn) is an essential trace element that is abundantly present in the brain. Despite its importance in normal brain functions, excess Zn is neurotoxic and causes neurodegeneration following transient global ischemia and plays a crucial role in the pathogenesis of vascular-type dementia (VD). We have investigated the molecular mechanisms of Zn-induced neurotoxicity using immortalized hypothalamic neurons (GT1–7 cells) and found that carnosine (β-alanyl histidine) and histidine (His) inhibited Zn^2+^-induced neuronal death. A DNA microarray analysis revealed that the expression of several genes, including metal-related genes (metallothionein and Zn transporter 1), endoplasmic reticulum (ER)-stress related genes (*GADD34*, *GADD45*, and *p8*), and the calcium (Ca)-related gene *Arc* (activity-related cytoskeleton protein), were affected after Zn exposure. The co-existence of carnosine or His inhibited the expression of *GADD34*, *p8*, and *Arc*, although they did not influence the expression of the metal-related genes. Therefore, ER-stress and the disruption of Ca homeostasis may underlie the mechanisms of Zn-induced neurotoxicity, and carnosine might be a possible drug candidate for the treatment of VD.

## 1. Introduction

The prevalence of senile dementia, which is a serious problem in a rapidly aging world, increases with age. Approximately 25% of all elderly individuals are affected by the disease. In 2012, more than 3 million people in Japan were estimated to be affected by senile dementia, and the number continues to grow annually. Senile dementia is mainly divided into Alzheimer’s disease (AD) and vascular-type dementia (VD). VD is a degenerative cerebrovascular disease, and its risk factors include age, the male gender, diabetes, and high blood pressure. The most common type of VD is caused by a series of small strokes or ischemia [1]. Following transient global ischemia or stroke, the interruption of blood flow and the resulting oxygen-glucose deprivation induces long-lasting membrane depolarization and an excessive release of glutamate into synaptic clefts. Thereafter, the excess glutamate causes an overstimulation of its receptors, namely *N*-methyl-d-aspartate (NMDA)-type receptors, amino-3-hydroxy-5-methyl-4-isoxazolepropionic acid (AMPA)-type receptors, and kainate-type receptors. Finally, Ca^2+^ dyshomeostasis, which involves the entry of large quantities of Ca^2+^ into glutamate-responsive neurons, triggers the delayed death of vulnerable populations of neurons, such as pyramidal neurons in the hippocampus—an area associated with learning and memory. Thereafter, the development of an infarct and subsequent cognitive dysfunction mark the pathogenesis of VD in elderly people. Approximately 30% of stroke patients show symptoms of dementia within 3 months of the initial stroke [2].

Increasing evidence has suggested that zinc (Zn) is central to ischemia-induced neuronal death and, finally, to the pathogenesis of VD [3]. In ischemia conditions, a considerable amount of Zn (up to 300 μM) is co-related with glutamate into synaptic clefts by membrane depolarization. We have found that GT1–7 cells, which are immortalized hypothalamic neurons, are more vulnerable to Zn than other neuronal cells are [4,5]. Zn causes the apoptotic death of GT1–7 cells in a dose-dependent and time-dependent manner. Mellon *et al.* originally developed GT1–7 cells by genetically targeting tumorigenesis in mouse hypothalamic neurons [6]. The cells possess neuronal characteristics, such as the extension of neurites and the secretion or the expression of several neuron-specific proteins or receptors. Additionally, GT1–7 cells either lack, or possess low levels of, ionotropic glutamate receptors and do not exhibit glutamate toxicity [7]. These properties make the GT1–7 cell line an excellent model system for the investigation of Zn-induced neurotoxicity. Furthermore, we developed a screening system with GT1–7 cells with substances that protect neurons against Zn, based on the idea that such substances may be potential candidates for the treatment of VD [8–11]. Among the various food products or agricultural products we tested, we found that carnosine (β-alanyl histidine) is markedly effective in preventing the neuronal death induced by Zn [12]. Carnosine has previously been reported to be effective in the treatment of other neurodegenerative diseases, including AD and prion disease, or aging-related disorders, including cataracts [13–15].

In this article, we review the current understanding of the molecular mechanisms of Zn-induced neurotoxicity and the link between Zn and the pathogenesis of VD. We also review the molecular mechanisms underlying the protective effects of carnosine in preventing neuronal death induced by Zn.

## 2. Zinc and Vascular-Type Dementia

### 2.1. Zinc-Induced Neurodegeneration after Ischemia

Zn is an essential trace element for most organisms. It plays important roles in various physiological functions such as in mitotic cell division, immune system activity, the synthesis of proteins and nucleic acids, and as a co-factor of more than 300 enzymes or metalloproteins [16]. Recent studies have revealed that Zn signaling plays crucial roles in various human biological systems [17]. Zn deficiency in human childhood is known to cause dwarfism, the retardation of mental and physical development, immune dysfunction, and learning disabilities [18].

The human body contains approximately 2 g of Zn, mostly in the testis, muscle, liver, and brain tissues. In the brain, Zn is found at the highest concentrations in the hippocampus, amygdala, cerebral cortex, thalamus, and olfactory cortex [19]. The total Zn content of the hippocampus has been estimated to be 70–90 ppm (dry weight). Although some Zn in the brain binds firmly to metalloproteins or enzymes, a substantial fraction (approximately 10% or more) either forms free Zn^2+^ or loosely bound Zn, and it is histochemically detectable with staining with chelating reagents. This chelatable Zn is stored in the presynaptic vesicles of specific excitatory glutamatergic neurons, and it is secreted from those vesicles into synaptic clefts, along with glutamate, during neuronal excitation. Recent studies have suggested that secreted Zn^2+^ plays crucial roles in information processing, synaptic plasticity, learning, and memory. Indeed, Zn^2+^ has been shown to be essential in the hippocampus for the induction of long-term potentiation, a form of synaptic information storage that has become a well-known paradigm for the mechanisms underlying memory formation [20].

**Figure 1:**
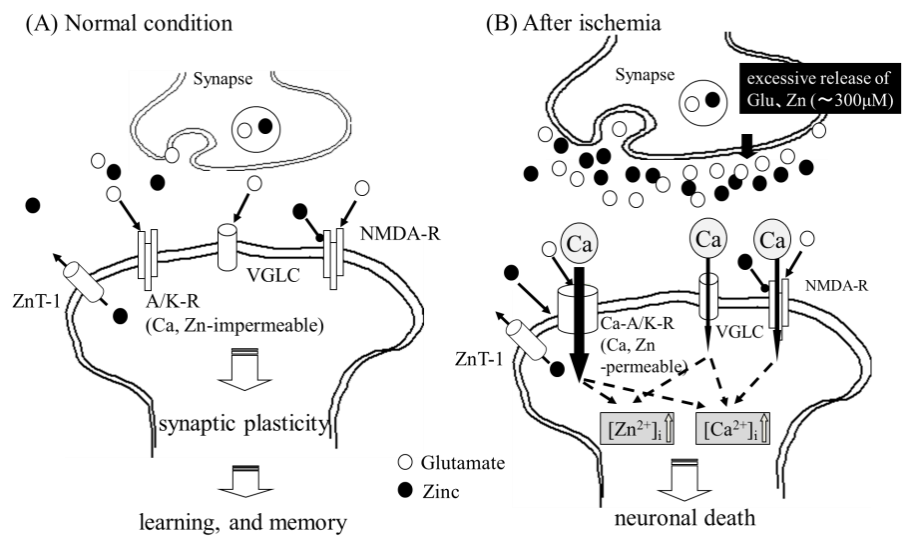
Zn in normal or pathological conditions in the brain. Under normal conditions (**A**), neuronal excitation causes the release of glutamate and Zn. Zn regulates postsynaptic excitability by binding to NMDA-type glutamate receptors (NMDA-R). However, under pathological conditions, such as ischemia (**B**), oxygen-glucose deprivation induces the release of excess glutamate, as well as Zn, into the synaptic cleft. Excess Zn enhances the expression of Ca-AMPA/kainate channels (Ca-A/K-R), and it is translocated through the Ca-A/K-R or through other pathways, such as voltage-gated l-type Ca^2+^ channels (VGLC), into the target neuron, where Zn acts to inhibit various enzymes, inhibit mitochondrial respiration, cause energy depletion, and produce ROS. Excess glutamate induces an increase in intracellular Ca^2+^ levels in the target neuron. Increased levels of intracellular Ca^2+^ then trigger various apoptotic pathways, such as those involving the activation of calpain or caspases, or other enzymatic pathways related to apoptosis; ultimately, this leads to neuronal death.

However, despite its importance, excess Zn is neurotoxic and has been implicated in neurodegenerative disease. Zn is shown in normal and ischemic conditions in the brain in Figure 1. In ischemic conditions, a considerable amount of Zn (up to 300 μM) is associated with glutamate in synaptic clefts due to membrane depolarization. Zn causes the apoptotic death of primary cultured cortical neurons. Furthermore, chelatable Zn reportedly moves from presynaptic terminals into postsynaptic neuronal cell bodies. An increase in intracellular Zn^2+^ levels ([Zn^2+^]_i_), namely, Zn translocation, occurs in vulnerable neurons in the CA1 or CA3 regions of the hippocampus prior to the onset of delayed neuronal death after transient global ischemia [21]. This Zn translocation has been reported to enhance the appearance of infarcts. The administration of calcium ethylenediaminetetraacetic acid (Ca-EDTA), a membrane-impermeable chelator that chelates cations other than Ca, has been shown to block the translocation of Zn, protect hippocampal neurons after transient global ischemia, and reduce infarct volume [22]. Thus, Zn translocation has been recognized as the primary event in the pathway of Zn-induced neuronal death. Sensi *et al*. have observed temporal changes in [Zn^2+^]_i_ in cultured cortical neurons with a Zn-sensitive fluorescent dye; those results revealed that at least 3 major routes of Zn^2+^ entry have been identified: Voltage-gated Ca^2+^ channels (VGLG), NMDA-type glutamate receptors, and AMPA/kainate-type glutamate receptors (A/K-R). Although the NMDA-type glutamate receptors are present in most neurons, the permeability of Zn^2+^ and Ca^2+^ through AMPA/kainate channels is greater than that of NMDA-type glutamate receptor channels [23].

In normal conditions, most hippocampal neurons express AMPA receptors with GluR2 subunits, which are poorly permeable to divalent cations, including Ca^2+^ and Zn^2+^(A/K-R). However, under ischemic conditions, an acute reduction in the expression of the GluR2 subunit occurs, and neurons possess specific types of AMPA receptors in which the channels are directly Ca^2+^ permeable (Ca-AMPA/kainate channels; Ca-A/K-R) [24]. The appearance of Ca-A/K-R causes an increased permeability of Ca^2+^ and enhances the toxicity. Therefore, the expression of Zn^2+^-permeable Ca-A/K-R and the entry of Ca^2+^ and/or Zn^2+^ through the channels are involved in delayed neuronal death after ischemia. Considering that Ca-EDTA, a Zn chelator, attenuates the ischemia-induced downregulation of the GluR2 gene [22], Zn has also been implicated in the transcriptional regulation of Ca-A/K-R.

These results strongly implicate Zn as a key player in delayed neuronal death after transient global ischemia, a process that might be involved in the pathogenesis of VD [25,26]. The accumulation of Zn has also been observed following head trauma and epilepsy [27], implying that Zn neurotoxicity might underlie the pathological mechanisms of various injuries. Moreover, the disruption of Zn homeostasis has also been implicated in other neurodegenerative diseases, including AD [28–30], prion disease [31], amyotrophic lateral sclerosis [32], and Wilson’s disease [33]. Thus, Zn might play a role like that of Janus, who is the ancient Roman god of doorways and who has 2 different faces, in the brain: Both Zn depletion and excess Zn cause severe damage to neurons.

### 2.2. Molecular Mechanism of Zn-Induced Neuronal Neath: GT1–7 Cells as an *In Vitro* Model System

Understanding the molecular mechanisms of Zn-induced neuronal death is of great importance for the treatment of VD. Numerous studies have been undertaken in order to elucidate the mechanisms of Zn-induced neuronal death. To this end, many researchers have investigated Zn neurotoxicity *in vitro* mainly by using primary cultured neurons from rat cerebral cortex or hippocampus [34] or PC-12 cells, a pheochromocytoma cell line [35]. However, the roles of Zn are highly complex. For example, Zn has been shown to inhibit NMDA-type glutamate receptors and to regulate the excitability of glutamatergic neurons, which are toxic to neurons. Therefore, distinguishing the effects of Zn and glutamate in neuronal cells that possess glutamate receptors has proven difficult.

**Figure 2:**
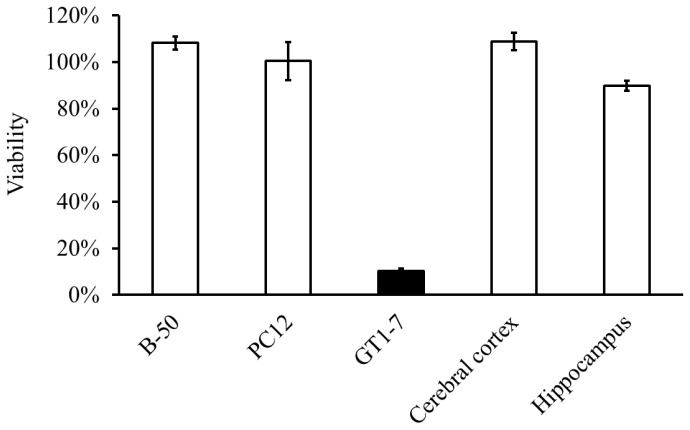
Apoptotic death of GT1–7 cells after exposure to Zn. Viability of various neuronal cells after exposure to Zn. Cultured neuronal cells (GT1–7 cells, PC-12 cells, B-50 cells (a neuroblastoma cell line), primary cultured neurons of the rat cerebral cortex, and primary cultured neurons of the rat hippocampus) were administered 50 μM of Zn. After 24 h, cell viability was analyzed by the WST-1 method.

We found that GT1–7 cells, which are immortalized hypothalamic neurons, are much more sensitive to Zn than other neuronal cells are [4,5]. Figure 2 shows the viability of GT1–7 cells, PC-12 cells, B-50 cells (a neuroblastoma cell line), and primary cultured neurons derived from the rat cerebral cortex or hippocampus following exposure to Zn. Zn caused the apoptotic death of GT1–7 cells in a dose-dependent and time-dependent manner. The degenerated GT1–7 cells are terminal deoxynucleotidyl transferase-mediated biotinylated UTP nick-end labeling (TUNEL)-positive and exhibit DNA fragmentation [4,5].

The GT1–7 cells, which were developed by genetically targeting the tumorigenesis of mouse hypothalamic neurons, possess a number of neuronal characteristics, such as the extension of neurites, the secretion of gonadotropin-releasing hormone (GnRH), and expression of neuron-specific proteins or receptors including microtubule-associated protein 2 (MAP2), tau protein, neurofilament, synaptophysin, GABA_A_ receptors, dopamine receptors, and l-type Ca^2+^ channels [6]. Additionally, GT1–7 cells either lack or possess low levels of ionotropic glutamate receptors and do not exhibit glutamate toxicity [7]. These properties make the GT1–7 cell line an excellent model system for the investigation of Zn-induced neurotoxicity.

### 2.3. Implication of Ca Dyshomeostasis in Zn-Induced Neuron Death

We investigated the detailed characteristics and mechanisms of Zn-induced death in GT1–7 cells. Previously, we tested the effects of the administration of various pharmacological agents prior to the Zn treatment of GT1–7 cells. We showed that the administration of sodium pyruvate, an energy substrate, significantly inhibited the Zn-induced death of GT1–7 cells [4]. The results were consistent with findings of other studies that used primary cultured cortical neurons, oligodendrocyte progenitor cells, or retinal cells. Furthermore, the administration of pyruvate attenuated neuronal death after ischemia *in vivo* [36]. Shelline and his colleagues have reported that Zn exposure decreases the levels of NAD^+^ and ATP in cultured cortical neurons and that treatment with pyruvate restores the NAD^+^ levels [37]. An imaging study that used a Zn-sensitive fluorescent dye and a mitochondrial marker revealed that Zn is localized within mitochondria. Zn has been reported to inhibit various mitochondrial enzymes and the intracellular trafficking of mitochondria. It has also been reported that Zn produces reactive oxygen species and causes oxidative damage resulting from mitochondrial impairments. Therefore, energy failure and the inhibition of glycolysis in mitochondria may be involved in Zn neurotoxicity [38].

**Figure 3:**
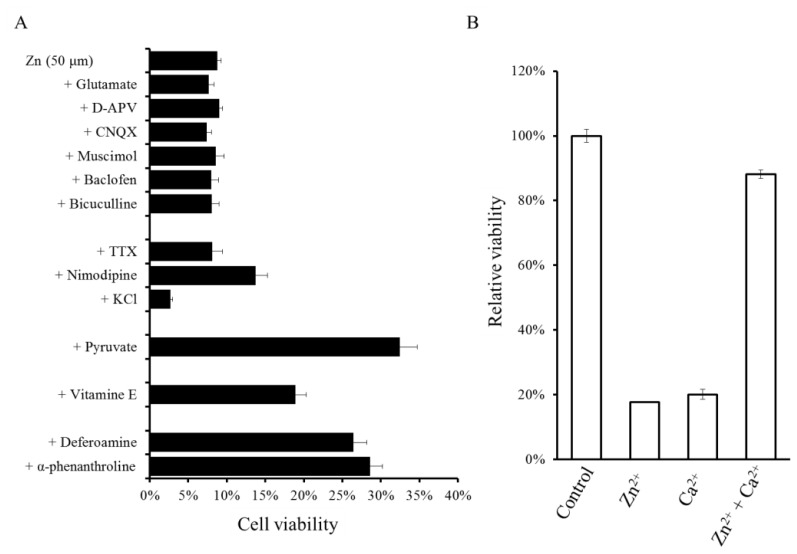
Effects of various pharmacological substances on the Zn-induced death of GT1–7 cells. (**A**) GT1–7 cells were exposed to 50 μM of Zn^2+^ with agonists or antagonists of neurotransmitters [glutamate, d-APV (d-2-amino-5-phosphonovalerate), CNQX (6-cyano-7-nitroquinoxaline-2,3-dione), muscimol, baclofen, bicuculline], GABA channel blockers [TTX (tetrodotoxin), nimodipine], *etc.* (**B**) The relative viability of GT1–7 cells exposed to Zn^2+^ with or without Ca^2+^.

Neither antagonists nor agonists of excitatory neurotransmitters [D2-amino-5-phosphonovalerate (APV), glutamate, and 6-cyano-7-nitroquinoxaline-2,3-dione(CNQX)], or those of inhibitory neurotransmitters (bicuculline, muscimol, baclofen, and GABA) attenuated the viability of GT1–7 cells after Zn exposure (Figure 3). Our findings in GT1–7 cells were inconsistent with previous studies that found that agonists of glutamate receptors, such as NMDA or AMPA, enhance Zn-induced neurotoxicity in cultured cortical neurons [39].

The co-exposure of GT1–7 cells to various metal ions with Zn has shown the involvement of other metal ions in Zn neurotoxicity [40]. The equivalent molar addition of Al^3+^ and Gd^3+^ significantly inhibited Zn-induced neuronal death. Moreover, the overloading of Ca^2+^ and Mg^2+^ inhibited the Zn-induced death of GT1–7 cells, and Zn protected GT1–7 cells from the neurotoxicity induced by Ca^2+^ overload and *vice versa* (Figure 3B).

**Figure 4:**
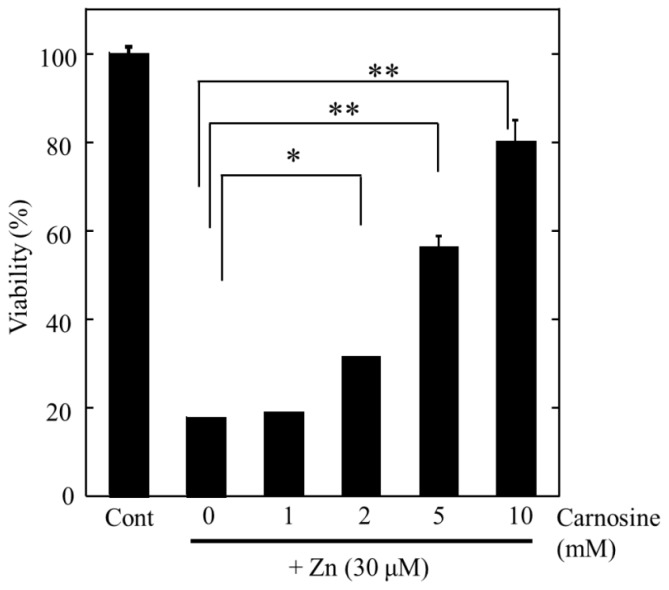
Protective activity against the Zn-induced neurotoxicity of carnosine and its stability in culture media. The protective activity of carnosine on Zn-induced death in GT1–7 cells. ZnCl_2_ (30 μM) was pre-administered to the GT1–7 cells. After 24 h, the viability of the GT1–7 cells was compared with the co-administration of various concentrations of carnosine. The data are presented as means ± S.E.M., *n* = 6. * *p* < 0.01, ** *p* < 0.005.

Considering the implication of Zn in transient global ischemia, substances that protect against Zn-induced neuronal death could be potential candidates for the prevention or treatment of neurodegeneration following ischemia and ultimately provide a lead for treatments for VD. We examined the potential inhibitory effects of various agricultural products, such as vegetable extracts, fruits extracts, and fish extracts, and found that extracts from eel muscles significantly protected against Zn-induced neurotoxicity [9]. Finally, we determined that carnosine, a small hydrophilic peptide abundant in eel muscles, protected GT1–7 cells from Zn-induced neurotoxicity in a dose-dependent manner (Figure 4).

Based on these findings, we investigated the molecular mechanisms underlying Zn-induced neurotoxicity. Our results suggested that Ca dyshomeostasis may be involved in the mechanisms of Zn-induced neurotoxicity. It has also been reported that Zn neurotoxicity in PC12 cells is attenuated by an l-type Ca^2+^-channel blocker, nimodipine, and enhanced by an l-type Ca^2+^ channel activator, S(−)-Bay K 8466 [35]. Additionally, Zn neurotoxicity is attenuated by aspirin, which prevents Zn^2+^ entry through voltage-gated Ca^2+^ channels [41]. In addition to this issue, we employed a high-resolution multi-site video imaging system with fura-2 as the cytosolic-free Ca reporter fluorescent probe in order to observe the temporal changes in [Ca^2+^]_i_ after exposure to Zn [5]. Zn caused increased [Ca^2+^]_i_ among GT1–7 cells after 3–30 min of exposure [5]. A detailed analysis of Zn-induced [Ca^2+^]_i_ revealed that the pretreatment of Al^3+^ significantly blocked the Zn-induced [Ca^2+^]_i_ increases. Thus, it is possible that Al^3+^, a known blocker of various types of Ca^2+^ channels, might attenuate Zn-induced neurotoxicity by blocking Zn-induced increases in [Ca^2+^]_i_.

### 2.4. Altered Gene Expression during Zinc-Induced Neurotoxicity

**Figure 5:**
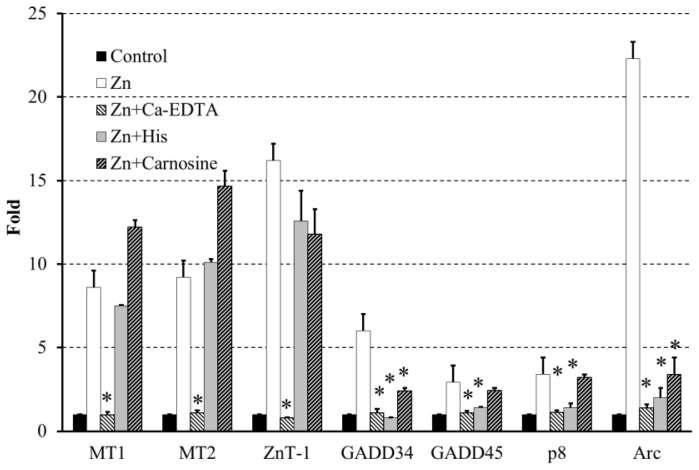
Effects of carnosine and His on Zn-induced gene expression. GT1–7 cells were exposed to 50 μM of ZnCl_2_ for 6 h in the presence or absence of Ca-EDTA (0.5 mM), His (1.0 mM), or carnosine (2.0 mM). The expression of various genes were analyzed by RT-PCR and the gene expression levels were normalized against β-actin. The data are expressed as means ± S.E.M., *n* = 3. * *p* < 0.01 *vs*. Zn.

Moreover, we investigated the alterations in gene expression during Zn-induced neurotoxicity with a DNA microarray and RT-PCR [42]. The expression of several genes, including metal-related genes, such as metallothionein (*MT*)*-1*, *MT-2*, and the Zn transporter 1 (*ZnT-1*) were induced by Zn exposure. Metallothioneins are intracellular polypeptides with a remarkable ability to bind metallic ions. *MT1* and *MT2* play roles in the detoxification of heavy metals. Zn-specific membrane transporter proteins (Zn transporters) also control Zn homeostasis; they facilitate Zn influx during deficiencies and efflux during Zn excess. Thus, these proteins are upregulated by increases in [Zn^2+^]_i_. (see Zn in Figure 5). Furthermore, our investigation into the changes in gene expression during Zn-induced neuronal death revealed an upregulation of several genes, including ER stress-related genes [growth-arrest DNA damage (*GADD*)*34*, *GADD45*, and *p8*) and the Ca^2+^-related gene *Arc* (activity-related cytoskeleton protein) [42]. These findings are important considering the involvement of Ca^2+^ homeostasis in Zn-induced neurotoxicity. It is widely accepted that the ER regulates the levels of [Ca^2+^]_i_ and that ER stress causes the apoptotic death of various cells by the accumulation of unfolded or misfolded proteins [43]. Emerging evidence has implicated ER stress in the pathogenesis of various neurodegenerative diseases, including AD, Parkinson’s disease, prion diseases, and ischemia-induced neurodegeneration [44,45]. In particular, *GADD34* and *GADD45* are the genes of the sensor proteins of ER stress, and they are induced by DNA damage and have been implicated in DNA repair and tumorigenesis [46]. The *p8* mRNA is induced in response to divergent stress, and it might be involved in tumorigenesis [47]. In addition, *Arc* is a gene that encodes a protein that exists in dendrites and plays crucial roles in synaptic plasticity and memory consolidation. *Arc* expression is induced by the increased neuronal activity that occurs in response to learning and by brain-derived neurotrophic factor (BDNF) [48].

The coexistence of Ca-EDTA abolished the effects of Zn in the expression of these genes (see Zn + Ca-EDTA in Figure 5). Meanwhile, the addition of carnosine and histidine (His) did not influence the upregulation of *MT1*, *MT2*, or *ZnT-1* during Zn-induced neurotoxicity (see Zn + Carnosine, Zn + His in Figure 5). However, the coexposure of GT1–7 cells to carnosine and His with Zn inhibited the expression of *GADD34*, *GADD45*, *p8* and *Arc* induced by Zn exposure (Figure 5). These attenuations of carnosine and His on the Zn-induced expression of these genes may affect ER stress-related or *Arc*-related apoptotic pathways.

## 3. Hypothesis Regarding Zn-Induced Neurotoxicity

**Figure 6:**
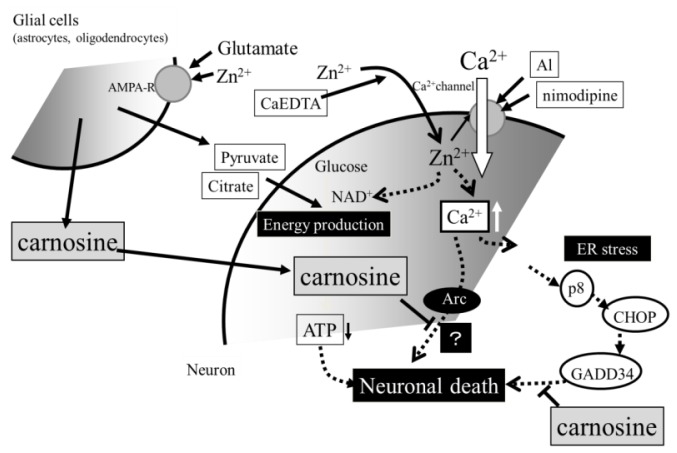
Hypothetical schematic of the molecular mechanisms of the protective effects of carnosine in preventing the neuronal death induced by Zn. Carnosine is synthesized in glial cells and is secreted in response to stimulation by glutamate and Zn; it protects neurons from Zn neurotoxicity. Carnosine inhibits the expression of ER stress-related genes and *Arc*, which are induced by Zn exposure. It is plausible that carnosine may be transported into cell bodies, where it can inhibit ER stress-related and/or *Arc*-related apoptotic pathways activated by Zn.

Collectively considering the accumulated evidence presented in this paper, we inferred a scheme for Zn neurotoxicity and the role of carnosine (Figure 6). In normal conditions, neuronal excitation causes the release of glutamate and Zn. However, Zn regulates postsynaptic excitability by binding to NMDA-type glutamate receptors. Zn in the synaptic clefts is either taken up, absorbed, or bound to the carnosine released from glial cells by the stimuli of glutamate and Zn. This feedback pathway of carnosine-Zn protects neurons against both glutamate toxicity and Zn toxicity. However, in pathological conditions, such as ischemia, oxygen-glucose deprivation induces the release of excess glutamate, as well as Zn, into synaptic clefts (Figure 1B). Excess Zn enhances the expression of Ca-A/K-R, and it is translocated through the Ca-A/K-R or through other pathways into the target neuron, where Zn acts to inhibit various enzymes, inhibit mitochondrial respiration, cause energy depletion, and/or produce reactive oxygen species. Excess glutamate induces an increase in the intracellular Ca^2+^ levels in the target neuron. Increased levels of intracellular Ca^2+^ then trigger various apoptotic pathways, such as those involving the activation of calpain or the activation of caspases, or other enzymatic pathways related to apoptosis; ultimately, this leads to neuronal death. Zn also influences intracellular Ca^2+^ levels and enhances the effects of glutamate. Our results of the Zn-induced upregulation of *GADD34*, *GADD45*, *p8*, and *Arc* suggest the implication of some ER stress-related or *Arc*-related pathways in ischemia-induced neuronal death and the pathogenesis of VD. This hypothesis is supported by the findings that the expression of *GADD34*, *GADD45*, and *Arc* was induced after ischemia [49–51].

## 4. Conclusions

Our results suggest the involvement of Ca^2+^ homeostasis and the gene expression of ER-stress related genes or *Arc* in Zn-induced neurotoxicity. These findings are important considering that Ca^2+^ homeostasis is involved in other neurodegenerative diseases including Alzheimer’s disease, prion diseases, Parkinson’s disease, ALS *etc*. Interestingly, Zn is involved in the pathogenesis of these neurodegenerative diseases and acts as a contributor of the disease in one part, and as a protector in another part. Thus, Zn might play a role like that of Janus, an ancient Roman god of doorways with two different faces, in the brain. Therefore, our hypothesis may help the understanding of VD as well as other neurodegenerative diseases.

Our results also indicate the relevance of carnosine and His for consideration in the treatment of VD. Carnosine, which is a naturally occurring dipeptide, is commonly present in vertebrate tissues, particularly within the skeletal muscles and nervous tissues [15]. It is found at high concentrations in the muscles of animals or fish that exhibit high levels of exercise, such as horses, chickens, and whales. The concentrations of carnosine in the muscles of such animals have been estimated to be 50–200 mM, and carnosine is believed to play important roles in the buffering capacities of muscle tissue. During high-intensity anaerobic exercise, proton accumulation causes a decrease in intracellular pH, which influences various metabolic functions. The p*Ka* value of carnosine is 7.01, which is close to intracellular pH. Therefore, carnosine contributes to physicochemical nonbicarbonate buffering in skeletal muscles, and the administration of carnosine has been reported to induce hyperactivity in animals.

Carnosine reportedly has various functions, including anti-oxidant, anti-glycation, and anti-crosslinking functions, and it is considered to be an endogenous neuroprotective and anti-aging substance. Considering the advantageous properties of carnosine (relatively nontoxic, heat-stable, and water-soluble), dietary supplementation of carnosine might be an effective strategy for the prevention or treatment of neurodegenerative diseases, such as ischemia, VD, AD, and prion diseases. Corona *et al.* have reported that the supplementation of carnosine improved the learning abilities of Alzheimer’s model mice [13]. We have demonstrated that the neurotoxicity of the prion protein fragment is attenuated by Zn and carnosine [14]. Therefore, we applied for patents for carnosine as a drug for the treatment of VD or for slowing the progress of cognitive decline after ischemia (application No. 2006-145857; publication No. 2007-314467 in Japan) [12] and for His (application No. 2008-098675; publication No. 2009-249335 in Japan). Thus, further research into the role of Zn in neuronal injury and the significance of Zn and Ca homeostasis might lead to the development of new treatments for VD.

In the brain, carnosine also exists in the olfactory bulb [52]. We have developed a high-performance liquid chromatography system for analyzing and quantifying carnosine [10] and have confirmed that carnosine is abundant in the olfactory bulb in the rat brain. Although the physiological roles of carnosine in the olfactory bulb are still unclear, olfactory bulb neurons are less sensitive to damage after ischemia compared to hippocampal neurons, in spite of the accumulation of Zn. Furthermore, the contents of carnosine have been shown to vary during development [53], and the content of carnosine in muscle is decreased in aged animals [54]. Therefore, carnosine may play protective roles in Zn-induced neurodegeneration after ischemia in the olfactory bulb. It is plausible that carnosine may be transported into cell bodies, where it can inhibit several apoptotic pathways activated by Zn (Figure 6).
